# Liver sulfatide-reactive type II NKT cells recognize endogenous phospholipids

**DOI:** 10.1186/2051-1426-2-S3-P215

**Published:** 2014-11-06

**Authors:** Shingo Kato, Masaki Terabe, Jay A Berzofsky

**Affiliations:** 1 National Cancer Institute/National Institutes of Health, Bethesda, MD, USA

## 

NKT cells are a unique population of T cells that recognize lipid antigens presented by a nonclassical MHC-like molecule CD1d. There are two types of NKT cells, type I and type II. Our group previously showed that type I NKT cells enhance and type II NKT cells suppress anti-tumor responses, and that these two types of NKT cells cross-regulate each other. One of the defined antigens for type I NKT cells is alpha-galactosylceramide (aGC), and aGC-loaded CD1d tetramers are widely used to study them. Unlike conventional T cells, each subset of NKT cells recognizes distinct antigens. Sulfatide (3-o-sulfo-beta-D-galactosylceramide), an endogenous lipid, is the only lipid proven to be recognized by type II NKT cells in vivo. In addition, recently phosphatidylglycerol (PG) and phosphatidylinositol (PI), also endogenous lipids, were reported to be recognized by type II NKT cell hybridomas. So far, type II NKT cells and their antigens are much less well characterized than type I due to lack of widely available tools to study them. Our laboratory had developed a new method to create sulfatide-loaded CD1d tetramers, which could identify type II NKT cells. In this study, we compared phospholipid-reactive type II NKT cells and sulfatide-reactive type II NKT cells. PG-or PI-loaded CD1d-tetramer-reactive NKT cells were observed in liver mononuclear cells, and they were distinct populations from aGC-loaded CD1d-tetramer-reactive cells, indicating that there were PG- and PI-reactive type II NKT cells. Simultaneous staining with PG-, PI-, and sulfatide-loaded CD1d-tetramers indicated, surprisingly, that these three populations were largely identical, even though the phospholipids have structures quite distinct from that of sulfatide. For functional studies, the effect of these lipid antigens in cytokine production and regulating anti-tumor immunity was tested. IFN-gamma and IL-4 production by spleen cells was detected after stimulation with sulfatide in vitro, but not with PG or PI. In the murine colon cancer cell line CT26 lung metastases model, the anti-tumor response induced by aGC was reduced by simultaneous injection of sulfatide. In contrast, PG injection didn't affect the anti-tumor response induced by aGC. These findings suggest that although PG and PI, when loaded onto CD1d, bind the same cells as do sulfatide-CD1d tetramers, their function in vitro and in vivo was distinct. Now we are further examining the activities of these phospholipids on type II NKT cells.

